# Genetic landscape of phospholamban cardiomyopathies

**DOI:** 10.3389/fcell.2025.1626242

**Published:** 2025-06-10

**Authors:** Elizabeth Vafiadaki, Ishita Chaudhari, Keisha Mireia Soliman, Aristides G. Eliopoulos, Evangelia G. Kranias, Despina Sanoudou

**Affiliations:** ^1^ Center of Basic Research, Biomedical Research Foundation of the Academy of Athens, Athens, Greece; ^2^ Clinical Genomics and Pharmacogenomics Unit, 4th Department of Internal Medicine, “Attikon” Hospital, Medical School, National and Kapodistrian University of Athens, Athens, Greece; ^3^ Department of Biology, Medical School, and GENOSOPHY S.A. spin-off company, National and Kapodistrian University of Athens, Athens, Greece; ^4^ Department of Pharmacology, Physiology and Neurobiology, University of Cincinnati College of Medicine, Cincinnati, OH, United States

**Keywords:** phospholamban, genetic variants, cardiomyopathies, precision medicine, genetic testing, genetic counseling

## Abstract

Phospholamban (PLN) is a key regulator of cardiac muscle contractility and has become a central focus in the study of cardiac disease. Variants in the PLN gene have been identified in patients with a wide range of phenotypes, including hypertrophic, dilated, and arrhythmogenic cardiomyopathies. The growing number of identified variants highlights the previously underappreciated role of PLN in cardiac pathophysiology. This review offers a comprehensive examination of the genetic landscape of PLN and evaluates the mechanistic effects of specific variants on cardiac function, aiming to uncover potential genotype-phenotype correlations. The rapidly expanding body of knowledge in this area is driving the development of advanced diagnostic and prognostic tools, as well as highly targeted therapeutic strategies. These advances underscore the importance of recognizing PLN’s role in cardiac disease and the value of genetic testing for accurate diagnosis, prognosis, effective management, and early risk prediction for family members.

## 1 Introduction

Cardiac muscle contraction and relaxation rely on tightly regulated calcium cycling. Contraction is initiated by Ca^2+^ influx through transmembrane voltage-gated calcium channels, which subsequently induces a sharp release of Ca^2+^ from the sarcoplasmic reticulum (SR) via ryanodine receptor activation. This rise in cytosolic Ca^2+^ enables its binding to troponin C, which displaces tropomyosin from actin filaments and promotes actin-myosin interaction and contraction (Bers, 2002). Relaxation is achieved primarily through the reuptake of Ca^2+^ into the SR by the sarcoplasmic/endoplasmic reticulum Ca^2+^-ATPase isoform 2a (SERCA2a), whose activity is controlled by phospholamban (PLN) (MacLennan and Kranias, 2003).

**FIGURE 1:**
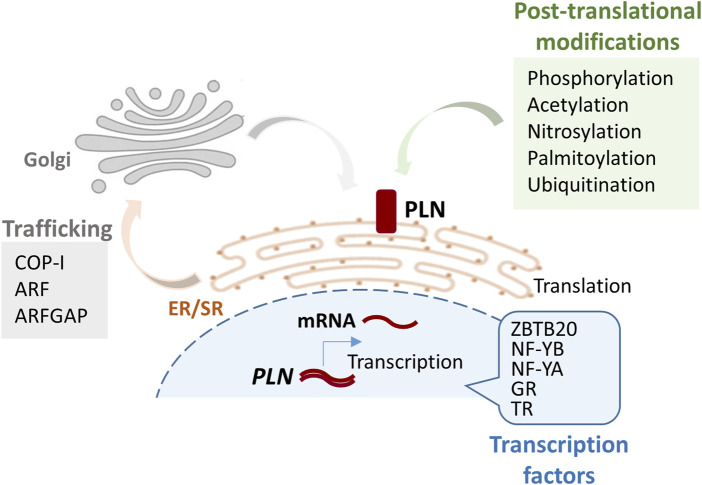
Schematic figure illustrating the pathway from *PLN* gene to protein and its localization at the SR membrane. Key factors and modifications involved in each step of the process are indicated. Post-translational modifications occur at lysine 3 (ubiquitination), cysteine 36 (palmitoylation), cysteines 41 and 46 (nitrosylation). GR, glucocorticoid nuclear receptor; TR, thyroid hormone receptor; COP-I, Coat Protein Complex I; ARF, ADP-ribosylation factor; ARFGAP, ADP-ribosylation factor GTPase-activating protein 1.

The *PLN* gene (OMIM: 172405) encodes a highly conserved protein that is primarily expressed in cardiac muscle and to a lesser degree in skeletal and smooth muscles (Fujii et al., 1991; Tada and Toyofuku, 1998; Tada et al., 1998). *PLN* is expressed early in development, indicating its essential role in cardiac physiology (Ganim et al., 1992; Simmerman and Jones, 1998). At the transcriptional level, *PLN* expression is known to be regulated by various transcription factors, including thyroid hormone receptors, glucocorticoid nuclear receptor, nuclear factor YA (NF-YA) and NF-YB, and the zinc finger protein ZBTB20 (Yabuki et al., 1998; Haghighi et al., 2008; Belakavadi et al., 2010; Ren et al., 2024), while post-transcriptionally its mRNA is stabilized by the RNA-binding protein Human Antigen R (HuR) (Hu et al., 2020). PLN is a 52-amino-acid transmembrane protein, localizing at the SR membrane and regulating SERCA2a activity (Tada and Toyofuku, 1998; Tada et al., 1998). Although information regarding trafficking of the protein to the SR is limited, the di-arginine motif in the cytoplasmic domain of PLN was shown to be required for retrograde trafficking between the Golgi to ER/SR compartments (Sharma et al., 2010) (Figure 1).

At the functional level, PLN regulates SERCA2a in a phosphorylation-dependent manner (MacLennan and Kranias, 2003; Kranias and Hajjar, 2017). When dephosphorylated, PLN inhibits SERCA2a by reducing its affinity for Ca^2+^, thereby delaying SR Ca^2+^ reuptake and relaxation. PLN phosphorylation at serine 16 (Ser16) by cAMP-dependent protein kinase (PKA) and at threonine 17 (Thr17) by Ca^2+^-calmodulin-dependent protein kinase (CaMKII) relieves this inhibition, leading to enhanced SR Ca^2+^ uptake and cardiac relaxation, particularly during β-adrenergic stimulation (MacLennan and Kranias, 2003). PLN acetylation, S-palmitoylation, and nitrosylation have emerged as additional post-translational modifications that modulate PLN function by influencing its membrane association, oligomerization, or interaction with SERCA2a, while ubiquitination regulates PLN protein levels (Froehlich et al., 2008; Irie et al., 2015; Zhou et al., 2015; Nakagawa et al., 2016; Keceli et al., 2019; Rogers et al., 2023; Mushala et al., 2024). Cytosolic Ca^2+^ levels also influence PLN-SERCA2a interactions, with elevated Ca^2+^ promoting PLN dissociation and SERCA2a activation (MacLennan and Kranias, 2003; Kranias and Hajjar, 2017). Structural studies have provided evidence to suggest that PLN adopts multiple conformational states which affect its inhibitory function on SERCA2a (Gustavsson et al., 2011; Gustavsson et al., 2013; Raguimova et al., 2020; Weber et al., 2021; Weber et al., 2024). In contrast to the initial, more simplistic, view that SERCA2a represents the sole binding partner of PLN, a more complex regulatory network has now been established through the identification of multiple and multifunctional binding proteins, signifying an essential contribution of PLN in cardiac function through multiple pathways (Vafiadaki et al., 2009; Haghighi et al., 2014; Kranias and Hajjar, 2017; Mattiazzi and Kranias, 2024).

The fundamental role of PLN in the heart is further corroborated by the detrimental effects *PLN* variants can have. To date, multiple pathogenic *PLN* variants have been identified in patients with a range of clinical cardiac phenotypes that include arrhythmogenic (ACM), dilated (DCM) and hypertrophic cardiomyopathy (HCM) (Kranias and Hajjar, 2017; Vafiadaki et al., 2023; Mattiazzi and Kranias, 2024).

According to the latest international guidelines and statements on genetic testing for cardiac disease, of the American Heart Association (AHA), the European Society of Cardiology (ESC), and the European Heart Rhythm Association (EHRA)/Heart Rhythm Society (HRS)/Asia Pacific Heart Rhythm Society (APHRS)/Latin American Heart Rhythm Society (LAHRS) (Musunuru et al., 2020; Wilde et al., 2022; Arbelo et al., 2023), *PLN* is in the recommended shortlist of genes to be tested in patients with a confirmed or suspected diagnosis of inherited HCM, DCM or ACM, or in individuals at high risk due to a previously identified pathogenic variant in their family.

The implementation of these guidelines is gradually leading to a marked increase in the number of novel *PLN* variants identified. Herein, we provide a comprehensive and critical overview of all human *PLN* variants known to date, including extensively characterized ones and/or described in the literature, as well as those reported solely across publicly available databases. The expanding research evidence on their respective pathogenetic mechanisms, is fueling the intense exploration of targeted precision medicine therapies, while genotype-phenotype correlations are geared towards guiding the development of diagnostically and prognostically valuable tools. Our study reveals a previously unanticipated extent of *PLN* contribution to cardiac disease worldwide, highlighting the need for heightened clinical awareness on the value of *PLN* genetic testing, as well as the interpretation and clinical integration of these results towards enhanced diagnosis, prognosis and clinical management of cardiomyopathy patients and their family members.

## 2 Published pathogenic *PLN* variants

The discovery of the first pathogenic *PLN* variants in 2003 (Haghighi et al., 2003; Schmitt et al., 2003), sparked interest in this gene and initiated a long path of research and discovery. Approximately half of the published pathogenic *PLN* variants have been characterized at the molecular/cellular/functional level, albeit the extent of characterization varies. The findings offer a deep understanding of the complex molecular mechanisms, implicated in PLN disease pathogenesis, and have led to the more effective treatment and clinical management of *PLN* cardiomyopathy patients (Hof et al., 2019). The key conclusions from these pathogenic *PLN* variants and their implications, are critically discussed by genomic location (coding or promoter region) and type of variation (deletion, missense, nonsense or insertion) (Table 1).

**TABLE 1:** *PLN* variants published to date and key phenotypic features. Classification is based on ClinVar’s 29 April 2025 release. VUS: variant of unknown significance, N/A: not available.

Type of variant	*PLN* variant	Classification	State	Clinical phenotype	Effect on protein	Reference
Deletion	c.40_42delAGA, p.Arg14del	Pathogenic	Heterozygote	Asymptomatic, ACM or DCM with arrhythmias	PLN structural changes, protein interaction aberrations, Ca^2+^ handling dysregulation, mitochondrial dysfunction, UPR activation, autophagy defects, SR disorganization and protein aggregation	Haghighi et al. (2003), van der Zwaag et al. (2012), Hof et al. (2019), Haghighi et al. (2006), Karakikes et al. (2015), Eijgenraam et al. (2020), Badone et al. (2021), Cuello et al. (2021), Feyen et al. (2021), Grote Beverborg et al. (2021), Haghighi et al. (2021), Raad et al. (2021), Dave et al. (2022), Vafiadaki et al. (2022), Cleary et al. (2023), Rogalska et al. (2023), Stege et al. (2023), Cleary et al. (2024), Foo et al. (2024), Maniezzi et al. (2024), Vafiadaki et al. (2024)
	c.95_98delTTAT, p.Phe32SerfsTer7	Pathogenic	Heterozygote	HCM	Predicted to result in nonsense-mediated mRNA decay but no evidence provided	Du et al. (2024)
Missense	c.23C>T, p.Thr8Ile	N/A	Not specified	DCM	Not known	Sousa et al. (2019)
	c.25C>T, p.Arg9Cys	Pathogenic	Heterozygote	DCM	Loss of function, increased propensity for oligomerization, decreased binding to SERCA2, decreased SERCA2 inhibition, aberrant Ca^2+^ handling, lack of phosphorylation, structural changes	Schmitt et al. (2003), Schmitt et al. (2009), Ha et al. (2011), Ceholski et al. (2012b), Abrol et al. (2015), Truszkowska et al. (2015), Fish et al. (2016), Nelson et al. (2018), Vicente et al. (2022), Weber et al. (2024)
	c.26G>T, p.Arg9Leu	Likely Pathogenic	Heterozygote	DCM	Loss of function, lack of phosphorylation, structural changes	Medeiros et al. (2011), Ceholski et al. (2012a), Ceholski et al. (2012b), Truszkowska et al. (2015), Weber et al. (2024)
	c.26G>A, p.Arg9His	Likely Pathogenic/VUS	Heterozygote	DCM	Loss of function, lack of phosphorylation	Medeiros et al. (2011), Ceholski et al. (2012a), Sousa et al. (2019), Weber et al. (2024)
	c.43G>A, p.Ala15Thr	Likely Pathogenic/VUS	Heterozygote	DCM	Gain of function, increased SERCA inhibition, reduced phosphorylation and dephosphorylation at Ser16	Pugh et al. (2014), Armanious et al. (2024)
	c.46T>G, p.Ser16Pro	N/A	Not specified	HCM	Not known	Walsh et al. (2017b)
	c.50C>A, p.Thr17Asn	N/A	Heterozygote	DCM	Not known	Mollanoori et al. (2018)
	c.53T>C; p.Ile18Thr	VUS	Not specified	HCM	Partial loss of function, reduced inhibition of SERCA, lack of PKA phosphorylation	Burns et al. (2017), Lopes et al. (2015), Armanious et al. (2024)
	c.61C>A, p.Pro21Thr	VUS	Heterozygote	DCM	Gain of function, increased SERCA inhibition, reduced phosphorylation and dephosphorylation at Ser16, increased helical structure	Pugh et al. (2014), Armanious et al. (2024)
	c.73C>T, p.Arg25Cys	Likely Pathogenic/VUS	Heterozygote	DCM, HCM and Bruganda syndrome	Gain of function, SERCA2 super-inhibition, elevated diastolic Ca^2+^, increased RyR Ser2814 phosphorylation	Behr et al. (2015), Liu et al. (2015), Lopes et al. (2015)
	c.121T>A, p.Cys41Ser	VUS	Not specified	HCM	Not known	Walsh et al. (2017b)
	c.145G>A, p.Val49Met	VUS	Heterozygote	HCM	Not known	Xu et al. (2015)
	c.152T>C, p.Leu51Pro	VUS	Not specified	HCM	Not known	Walsh et al. (2017b), Lopes et al. (2015)
Nonsense	c.4G>T, p.Glu2Ter	Pathogenic	Homozygote	DCM and severe heart failure	Loss of function, no PLN protein expression	Li et al. (2019)
	c.116T>G, p.Leu39Ter	Pathogenic/Likely Pathogenic	Heterozygote Homozygote	Asymptomatic hypertrophy, HCM or severe DCM with cardiac arrhythmias	Loss of function, no PLN protein expression, no SERCA2 inhibition	Haghighi et al. (2003), Landstrom et al. (2011), Medeiros et al. (2011), Sanoudou et al. (2015), Walsh et al. (2017a), Walsh et al. (2017b), Mazzone et al. (2024)
Insertion	c.9_10insA, p.Val4SerFsTer15	Pathogenic	Heterozygote	Wolf-Parkinson-White syndrome with unconfirmed diagnosis of cardiomyopathy	Frameshift and premature termination, produces protein of 17 amino acids but with only the initial 3 residues corresponding to PLN, no evidence provided	Truszkowska et al. (2015)
	c.37_38insA, p.Arg13LysfsTer7	N/A	Heterozygote	DCM	Frameshift alterations and premature termination after 7 amino acids but no evidence provided	Pugh et al. (2014)
	c.61_62insCT, p.Pro21LeufsTer19	N/A	Heterozygote	HCM	Frameshift alterations and premature termination after 19 amino acids but no evidence provided	Lopes et al. (2015)
	c.63_64dupTC, p.Gln22LeufsTer19	Pathogenic/Likely pathogenic/VUS	Not specified	HCM	Frameshift alterations and premature termination after 19 amino acids but no evidence provided	Walsh et al. (2017b)
	c.138dupT, p.Ile47TyrfsTer14	N/A	Not specified	HCM	Changes the last 6 amino acids of PLN and extends the protein by 8 residues but no evidence provided	Walsh et al. (2017b)
Promoter	−77 A>G	N/A	Heterozygote	HCM	Increased promoter activity	Minamisawa et al. (2003)
	−42 C>G	N/A	Heterozygote	HCM and healthy	Decreased promoter activity	Medin et al. (2007)
	−36 A>C	N/A	Heterozygote	DCM and healthy	Increased promoter activity	Haghighi et al. (2008)

### 2.1 Pathogenic coding *PLN* variants

The majority of published pathogenic *PLN* variants lie in the coding region, and they include missense and nonsense substitutions, as well as small deletions or insertions. While missense substitutions represent the most frequent type of variant, only a few have been well characterized.

#### 2.1.1 Pathogenic deletion variants

The *PLN* deletion c.40_42delAGA, p. Arg14del (or R14del) (OMIM: 172405.0003) has been the most extensively studied variant across clinical, cellular and molecular levels (Doevendans et al., 2019). It was originally identified in a large Greek family with DCM and arrhythmia symptoms (Haghighi et al., 2006) and has now been reported in >1,500 patients worldwide, with the vast majority being in the Netherlands (Hof et al., 2019). *PLN*-Arg14del has only been found in the heterozygous state, with patients exhibiting a spectrum of highly variable phenotypes that range from asymptomatic to ACM or DCM that may progress to heart failure or sudden cardiac death (Hof et al., 2019). *PLN*-Arg14del represents a prime example of the significant clinical impact that the in-depth characterization of a genetic variant can have.

Clinical research on *PLN*-Arg14del has demonstrated that these patients exhibit certain distinct clinical characteristics including low-voltage electrocardiograms (ECGs), negative T waves in left precordial leads and a high prevalence of malignant ventricular arrhythmias (van der Zwaag et al., 2012; van Rijsingen et al., 2014; Hof et al., 2019). These findings have been used for the development of multiple impactful clinical diagnostic and prognostic tools. Deep learning models have been developed that significantly improve ECG-based *PLN*-Arg14del automated detection of this rare group of patients (Lopes et al., 2021; Bleijendaal et al., 2021). At the prognostic level, multivariant clinical prediction algorithms have been aimed at identifying *PLN*-Arg14del patients at high risk of malignant ventricular arrhythmia, who should therefore be considered for primary prevention implantable cardioverter defibrillator (ICD) implantation (Verstraelen et al., 2021). The delineation of *PLN*-Arg14del as a distinct disease entity has increased the rate of *PLN*-Arg14del patient identification, leading to better clinical management. Furthermore, it enabled the formation of an international patient community and the establishment of the patient-led PLN Foundation (Kranias et al., 2018; Doevendans et al., 2019), which has since been actively facilitating and promoting PLN-Arg14del research towards finding a cure.

At the basic and translational research level, a multitude of *in vitro* and *in vivo* tools have been employed to decipher the PLN-Arg14del driven pathogenesis, including animal models ranging from zebrafish to mouse and patient-derived induced pluripotent stem cell cardiomyocytes (iPSC-CMs) (Haghighi et al., 2006; Karakikes et al., 2015; Eijgenraam et al., 2020; Badone et al., 2021; Cuello et al., 2021; Feyen et al., 2021; Grote Beverborg et al., 2021; Haghighi et al., 2021; Raad et al., 2021; Dave et al., 2022; Vafiadaki et al., 2022; Cleary et al., 2023; Rogalska et al., 2023; Stege et al., 2023; Cleary et al., 2024; Foo et al., 2024; Maniezzi et al., 2024; Vafiadaki et al., 2024). The results pinpoint defects in molecular mechanisms, including PLN structural changes, protein interaction aberrations, Ca^2+^ handling dysregulation, mitochondrial dysfunction, unfolded protein response (UPR) activation, autophagy defects, SR disorganization and protein aggregation [reviewed in (Vafiadaki et al., 2023; Stege et al., 2024; Deimen et al., 2022)]. Standard heart failure therapy involving eplerenone or metoprolol was shown to be ineffective in PLN-Arg14del heterozygous mice (Eijgenraam et al., 2020), underscoring the need for the development of novel therapeutic strategies targeting PLN-Arg14del. Excitingly, within 20 years since the discovery of this rare variant, a multitude of novel therapeutic approaches are being developed, including gene therapy, gene editing (CRISPR/Cas9 and TALENs), antisense oligonucleotides, and small molecules (Karakikes et al., 2015; Feyen et al., 2021; Grote Beverborg et al., 2021; Dave et al., 2022; Eijgenraam et al., 2022).

Recently, another pathogenic (ACMG criteria based classification: PVS1, PS2, PM2) deletion variant was reported in *PLN* (c.95_98del, p. Phe32SerfsTer7) in a heterozygous patient with complex clinical features due to dual diagnosis of cardiomyopathy and Perrault syndrome (Du et al., 2024). Notably, along with this *PLN* variant, a pathogenic variant was detected in Required for Meiotic Nuclear Division 1 homolog (*RMND1)* which might be responsible for the Perrault syndrome (Du et al., 2024). *PLN*-Phe32SerfsTer7 was predicted to disrupt the translational reading frame, leading to nonsense-mediated mRNA decay. However, there are no data available regarding the impact of this mutation on PLN protein expression levels, or its potential functional consequences in the cardiomyocytes.

#### 2.1.2 Pathogenic missense variants

The missense variant c.25C>T, p. Arg9Cys (OMIM: 172405.0001) (Schmitt et al., 2003) along with the nonsense variant c.116T>G, p. Leu39Term (OMIM: 172405.0002) [(Haghighi et al., 2003); see below] were the first *PLN* variants to be discovered in DCM patients, linking *PLN* genetic changes to cardiac disease. The familial *PLN*-Arg9Cys has only been encountered in heterozygosity and exhibits autosomal dominant inheritance (Schmitt et al., 2003; Truszkowska et al., 2015; Fish et al., 2016). Detailed analysis at the molecular, cellular and animal model levels has shown that PLN-Arg9Cys represents a loss-of-function variant, with a dominant-negative effect on wild-type PLN. It interferes with PKA-mediated PLN phosphorylation and consequently disrupts the regulatory function of PLN resulting in sustained SERCA2 inhibition, development of DCM, heart failure and premature death.

Recent studies on iPSC-CMs, recapitulated disease mechanisms associated with PLN-Arg9Cys, exhibiting aberrant Ca^2+^ handling, blunted β-adrenergic signaling, hypertrophy and fibrosis-related changes along with alterations in cellular metabolism and proteostasis disruption (Ceholski et al., 2018; Yu et al., 2024). Interestingly, exposing these cells to functional challenges exacerbated the PLN-Arg9Cys defects, leading to autophagic overload, structural remodeling and functional deficiencies (Yu et al., 2024). These research insights are guiding efforts towards the development of targeted therapeutic approaches for PLN-Arg9Cys carriers. Autophagy inducing agents tested on patient-derived PLN-Arg9Cys iPSC-CMs gave promising results, rescuing, at least in part, functional defects including structural remodeling, Ca^2+^ handling and contractile function (Yu et al., 2024).

In the same nucleotide position of *PLN* two more variants, c.26G>T, p. Arg9Leu and c.26G>A, p. Arg9His, have been identified, in heterozygosity, following mutation screening of Polish, Brazilian and Portuguese DCM patient cohorts (Medeiros et al., 2011; Truszkowska et al., 2015; Sousa et al., 2019). Similar to PLN-Arg9Cys, the Arg9Leu and Arg9His variant proteins do not get phosphorylated, most likely due to structural alterations in the region around the PKA phosphorylation site (Medeiros et al., 2011; Ceholski et al., 2012a). Interestingly however, only PLN-Arg9Leu failed to inhibit SERCA activity as, similarly with PLN-Arg9Cys, this variant results in altered hydrophobicity that impacts its function (Ceholski et al., 2012a; Ceholski et al., 2012b). In contrast, the inhibitory effect of Arg9His was indistinguishable from the wild-type PLN probably due to similar physicochemical properties of the substituted amino acid (Ceholski et al., 2012a).

A different heterozygous substitution, *PLN* c.73C>T, p. Arg25Cys, has been identified by multiple studies in patients presenting DCM and cardiac arrhythmias, HCM or Brugada syndrome patients with QRS duration (Behr et al., 2015; Liu et al., 2015; Lopes et al., 2015). Functional analysis by adenoviral overexpression in adult rat cardiomyocytes demonstrated that the PLN-Arg25Cys protein causes SERCA2 super-inhibition, resulting in suppressed Ca^2+^ kinetic parameters and depressed myocyte contractility (Liu et al., 2015). The molecular mechanisms associated with this included increased interaction of PLN-Arg25Cys with SERCA2a, most likely due to structural alterations (Liu et al., 2015; Nelson et al., 2018), leading to elevated diastolic Ca^2+^ and consequent activation of CaM kinase II (CaMKII). Although the latter did not affect PLN phosphorylation, it caused enhanced RyR phosphorylation at the Ser2814 site with consequent increases in SR Ca^2+^ leak which promoted arrhythmogenesis under stress conditions (Liu et al., 2015). These findings provided initial evidence to support the hypothesis that the increased PLN inhibitory function may impact both SR Ca^2+^ uptake and Ca^2+^ release, expanding our understanding of cardiomyocyte Ca^2+^ homeostasis regulatory mechanisms (Liu et al., 2015; Wagner et al., 2015).

Over the years, an increasing number of heterozygous missense *PLN* variants have been detected including c.23C>T, pThr8Ile; c.43G>A, p. Ala15Thr; c.46T>G, p. Ser16Pro; c.50C>A, pThr17Asn; c.53T>C; p. Ile18Thr; c.61C>A, p. Pro21Thr; c.121T>A, Cys41Ser; c.145G>A, p. Val49Met and c.152T>C, Leu51Pro (Pugh et al., 2014; Lopes et al., 2015; Xu et al., 2015; Burns et al., 2017; Walsh et al., 2017b; Mollanoori et al., 2018; Sousa et al., 2019). Among them, structural and functional evaluation has been pursued only for Ala15Thr, Pro21Thr, and Ile18Thr, albeit to a limited extent. Both former variants exhibited increased inhibition of SERCA activity and reduced rates of both PLN phosphorylation and dephosphorylation, whereas the latter variant caused reduced SERCA inhibition and no PKA phosphorylation (Armanious et al., 2024). While no structural changes were observed for Ala15Thr and Ile18Thr, the PLN Pro21Thr variant had increased helical structure, an alteration that could be correlated with its gain-of-function effect on SERCA activity (Armanious et al., 2024).

Overall, missense variants represent the most populated category of *PLN* variants. Their downstream molecular implications vary considerably, while in many instances they remain unknown. As the patient population carrying these variants increases, genotype-phenotype studies will be valuable to unveil diagnostically, prognostically and/or therapeutically relevant correlations.

#### 2.1.3 Pathogenic nonsense variants

The *PLN* nonsense variant c.116T>G, p. Leu39Term (also encountered in the literature as Leu39stop and Leu39X) was initially reported in a Greek family with DCM (Haghighi et al., 2003) but has since been identified in additional populations, with patients exhibiting clinical phenotypes ranging from asymptomatic hypertrophy to HCM or severe DCM with cardiac arrhythmias (Chiu et al., 2007; Landstrom et al., 2011; Medeiros et al., 2011; Alfares et al., 2015; Sanoudou et al., 2015; Walsh et al., 2017a; Walsh et al., 2017b; Mazzone et al., 2024). This familial *PLN* variant has been encountered in both heterozygous and homozygous state, with the existence of genetic modifiers potentially contributing in this wide phenotypic variability (Sanoudou et al., 2015). Examination of explanted cardiac tissue from a homozygote patient revealed more than 50% reduction in PLN mRNA levels and no detectable PLN protein, indicating that PLN-Leu39Ter is a null variant (Haghighi et al., 2003). These findings were corroborated by cellular studies following overexpression of recombinant PLN-Leu39Ter in HEK293 and adult rat cardiomyocytes which determined absence of stable PLN-Leu39Ter protein expression and consequent lack of SERCA2a activity inhibition (Haghighi et al., 2003). Recently, the absence of PLN-Leu39Ter protein was demonstrated to be due to its rapid proteosomal degradation following its translation (Rohner et al., 2021).

Another nonsense *PLN* variant, c.4G>T, p. Glu2Ter, has been identified in a 36 year old patient with DCM and severe heart failure (Li et al., 2019). This variant exhibited autosomal recessive inheritance. The mutation was present in homozygous state in the identified patient, while heterozygous carriers of the family had normal cardiac function. The p. Glu2Ter variant did not affect PLN expression at the mRNA level but it reduced PLN protein levels to about 50% in unaffected heterozygotes and abolished PLN protein expression in the homozygous patient (Li et al., 2019). Absence of the PLN protein was therefore proposed to be causative of the severe cardiac phenotype observed in this patient.

#### 2.1.4 Pathogenic insertion variants

Several *PLN* insertion variants have been reported so far, however, their functional significance has yet to been explored experimentally. All variants pertain a single or double base insertion that causes open reading frameshift changes with consequent inclusion of premature stop codon and protein termination. Among them, the single base insertion c.37_38insA, p. Arg13LysfsTer7, which causes frameshift alterations and premature termination after 7 amino acids, was identified in a patient with clinical diagnosis and familial history of DCM (Pugh et al., 2014). The single base insertion c.138dup, p. Ile47TyrfsTer14 was identified in an HCM patient (Walsh et al., 2017b). This variant occurs at the C-terminal end of the protein and would be expected to change the last 6 amino acids, removing the termination codon and extending the protein by 8 residues. Given that this insertion occurs within the transmembrane domain of PLN, a region that is critical both for its interaction with SERCA2 and its SR membrane localization (Kimura et al., 1996; Asahi et al., 1999; Abrol et al., 2014), expression of this protein variant would be expected to severely disrupt PLN function. Another single base insertion (c.9_10insA, p. Val4SerFsTer15) has been reported in a case with Wolf-Parkinson-White syndrome but with unconfirmed diagnosis of cardiomyopathy. As this insertion occurs close to the amino terminal sequence of the protein, it is expected to cause a frameshift and premature termination after 15 amino acids, leading to a protein of just 17 amino acids in length but with only the initial 3 residues corresponding to PLN (Truszkowska et al., 2015). Apart from these single base insertions, two cases of two base pair insertions (c.63_64dup, p. Gln22LeufsTer19 and c.61_62insCT, p. Pro21LeufsTer19) have been reported in patients with HCM (Lopes et al., 2015; Walsh et al., 2017b). Both variants were predicted to cause frameshift alterations and introduction of a premature termination after 19 amino acids. Collectively, while several insertion variants have been identified, it remains to be determined if PLN protein can be expressed in their presence, and if so, to delineate the impact on PLN function.

### 2.2 Non-coding pathogenic *PLN* variants

Only three variants in the promoter region of *PLN* have been published to date (−77 A>G, OMIM: 172405.0004, −42 C>G, OMIM: 172405.0005 and −36 A>C, OMIM: 172405.0006) (Minamisawa et al., 2003; Medin et al., 2007; Haghighi et al., 2008). They were all found in heterozygous state in patients with DCM (Haghighi et al., 2008) or HCM (Minamisawa et al., 2003; Medin et al., 2007). *In vitro* analysis in cell culture systems determined that the −77 A>G and −36 A>C variants increase PLN promoter activity by 50% and 24% respectively, while the presence of −42 C>G had the opposite effect causing nearly 50% decrease in promoter activity (Minamisawa et al., 2003; Medin et al., 2007; Haghighi et al., 2008). These changes may be due to alterations in transcription factor binding on the variant sequence, as indicated in the case of −36 A>C which resulted in enhanced binding of the glucocorticoid nuclear receptor (Haghighi et al., 2008). However, both the −42 C>G and the −36 A>C variants have also been detected in unaffected (up to the time of genetic testing) individuals (Medin et al., 2007). As a consequence, although one could speculate a tentative effect on PLN expression levels, the precise contribution of these non-coding variants in disease pathogenesis is currently unclear (Santos et al., 2009; Hirtle-Lewis et al., 2013).

## 3 *PLN* variants reported in genetic databases

Beyond the published *PLN* variants, the increasing awareness and application of cardiogenetic testing is leading to a rapidly expanding list of *PLN* variants in public repositories. Diving into this information can offer valuable insights. The ClinVar database (https://www.ncbi.nlm.nih.gov/clinvar/; search performed on 11th March 2025) has 145 entries of short (<50bps) *PLN* variants. Among them, the vast majority (n = 82) is listed as variants of unknown significance (VUS), 12 as pathogenic/likely pathogenic (P/LP) and 10 as conflicting (for 6 of which the conflict was between P/LP and VUS). In this list of *PLN* ClinVar P/LP/VUS/conflicting variants, the majority (n = 47) involves missense alterations, 12 frameshift and 3 nonsense. Interestingly, 29 UTR alterations are also listed, all of which are currently categorized as VUS (with the exception of 1 conflicting: VUS vs. benign), and 27 of which were detected in patients with either DCM or HCM. In some of these cases, the inheritance pattern of DCM in the family is described as dominant. The majority (n = 24) of the 29 *PLN* UTR alterations involved the 3′ UTR sequence, however, there is currently no experimental evidence regarding their contribution in disease pathogenesis. Although our ability to determine the clinical impact of variants in the 3′ UTRs of genes remains poor, it is well established that they comprise the bulk of noncoding sequences present in exomes, and are important for regulation of messenger RNA (mRNA) processing, stability, translation and subcellular localization (Mayr, 2019). Investigating the implications of these UTR variants at the molecular and clinical level could impact the design and interpretation of *PLN* genetic test results.

In addition to these short (<50 bps) *PLN* variants, copy number variants (CNVs) are also listed in the database (n = 12), including both copy number loss (n = 7) and copy number gain (n = 5). However, as these type of variants encompass large genomic regions (>1 kb), affecting multiple genes, their effect would be non-*PLN* specific and therefore difficult to interpret at the clinical level.

Similarly to the information available in ClinVar, gnomAD (https://gnomad.broadinstitute.org/) lists a large number of *PLN* variants beyond what is described in the literature (search performed on 11th March 2025). In specific, 193 variants are listed, of which only 47 are reported as overlapping with the *PLN* ClinVar variants. Notably, among the 193 variants, 65 involve missense changes, 10 frameshift, and strikingly, 95 involve UTR changes. Unlike the ClinVar data, most of the UTR variants in gnomAD are located in the 5′ UTR region (n = 56). Although the role of *PLN* UTR variants remains hypothetical and without experimental validation, such 5′ UTR variants are increasingly associated with cardiovascular disease in literature reports and investigated as therapeutic targets (Liang et al., 2017; Roberts et al., 2020; Soukarieh et al., 2022).

The predicted impact of many of these novel coding variants on PLN protein structure will require molecular and cellular approaches similar to those employed to date for published *PLN* variants. However, the even greater number of UTR variants, points to the need for additional research avenues that will delve into the under-recognized class of non-coding UTR variants, and characterize their tentative role in *PLN* cardiomyopathies’ pathogenesis, as well as their diagnostic value and therapeutic potential (Whiffin et al., 2020).

## 4 Discussion

**FIGURE 2:**
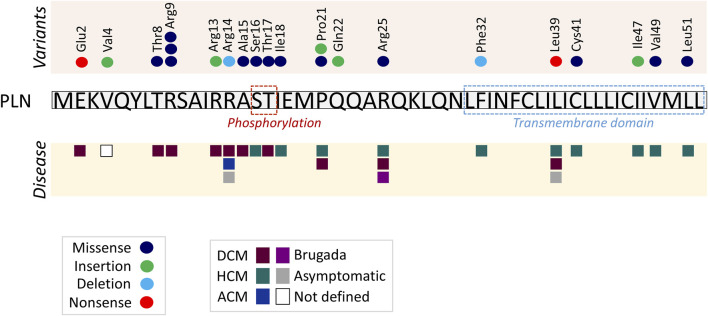
Diagrammatic representation of published *PLN* variants and associated clinical phenotypes. Each circle represents a different published PLN variant in the specific amino acid, and each square a different phenotype. Protein phosphorylation residues and transmembrane domain are indicated in dashed line boxes. PLN cytoplasmic residues 2–18 interact with a cytoplasmic fragment of SERCA2 while residues 20–30 and 31–52 bind to different transmembrane regions of SERCA2 (Kimura et al., 1996; Kimura et al., 1998; MacLennan et al., 1998; Asahi et al., 1999; Asahi et al., 2003; Toyoshima et al., 2003).

The present study has compiled information from all published *PLN* variants as well as in-depth analysis of the reported *PLN* variants in the ClinVar and gnomAD genetic databases, revealing the complex genetic contribution of *PLN* to cardiac disease. Among the rapidly increasing number of published *PLN* variants, the vast majority are missense, while nonsense variants appear to be rare (Figure 2). Several small insertions have been detected, all of which result in frameshift alterations that cause premature termination of protein. On the contrary, the small *PLN* deletions described cause either premature termination due to open reading frame alterations or single amino acid residue deletion, as in the case of PLN-Arg14del. The same trend of variant type frequencies is observed in the P/LP/VUS *PLN* variants submitted to the ClinVar and gnomAD databases.

The majority of published pathogenic variants are located within the N-terminal region of PLN (amino acids 1–30; n = 16), and fewer at the transmembrane domain (amino acids 31–52; n = 6) (Figure 2). A similar trend, yet less prominent, is observed among the P/LP/VUS/Conflicting ClinVar (40 N-terminal vs. 27 transmembrane) and the non-synonymous gnomAD (45 N-terminal vs. 31 transmembrane) *PLN* variants.

Furthermore, although there does not appear to be a distinct “variant hotspot,” specific residues may be more prone to mutagenesis. For example, multiple different alterations have been observed at a single amino acid position (Arg9), namely, Arg9Cys, Arg9Leu and Arg9His. Intriguingly, despite the fact that substitution occurs at the same position, not all variants have the same impact on the protein. This may be directly associated with physicochemical properties of the substituted amino acid and/or structural changes ensued. In specific, hydrophobic substitutions, such as Arg9Cys and Arg9Leu, increase propensity of oligomerization and eliminate SERCA inhibition while the aromatic substitution Arg9His does not impact SERCA activity (Ceholski et al., 2012a; Ceholski et al., 2012b; Abrol et al., 2015). According to a recent NMR spectroscopy study, the extent of PLN inhibition on SERCA activity correlates with the tilt angle of PLN’s transmembrane domain and PLN-Arg9Cys, Arg9Leu and Arg9His variants were shown to impact this on varying degrees (Weber et al., 2024). Nevertheless, unresponsiveness to β-adrenergic stimulation due to lack of phosphorylation of all three variants represents a common mechanism contributing to disease (Ceholski et al., 2012a; Ceholski et al., 2012b; Abrol et al., 2015). Similarly to the published Arg9 variants, three or more different variants have been submitted to ClinVar and gnomAD for the PLN amino acid positions Ile12, Arg25, Gln29, Ile40, Ile47, Met50 and Leu51, all of which are missense or frameshift.

**FIGURE 3:**
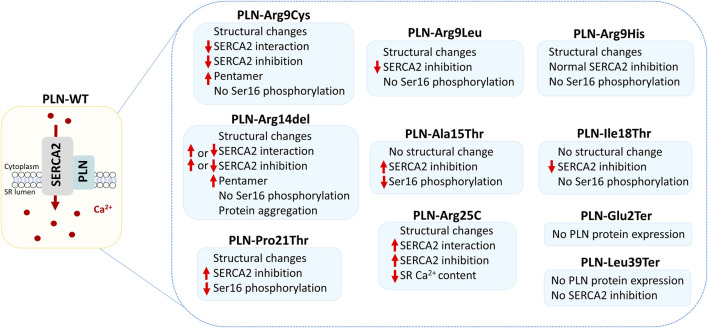
Functional effects of *PLN* variants. The schematic depicts the PLN and SERCA2 protein complex at SR membrane, where it regulates Ca^2+^ homeostasis and cardiac function. Alterations mediated by the different PLN variants are shown, based on currently available experimental evidence.

The fact that *PLN* variants lead to multiple forms of cardiomyopathies, along with the various clinical manifestations associated with single variants, such as Arg14del, Arg25Cys and Leu39Ter, suggest the absence of a clear genotype-phenotype correlation. However, close examination of the location of the variants suggests a potential topological correlation with the type of disease ensued. In specific, variants causing DCM are only occurring in the N-terminal and cytoplasmicc region of PLN, while HCM-causing variants are primarily found towards the C-terminal and within the transmembrane domain of PLN (Figure 2). An exception to this, are variants on residues Pro21, Arg25 and Leu39 that have been described in both DCM and HCM patients. In these cases, genetic or non-genetic factors, as discussed below, may act as modifiers of disease. While the contribution of such modifiers cannot be excluded, additional contributing mechanisms may underlie the intriguing topological distinction between the type of disease (DCM vs. HCM). In particular, the location of each variant may have a differential impact on PLN functional properties. This may include alterations in PLN structure, phosphorylation and/or protein interactions (Figure 3). Indeed, based on evidence from PLN-Arg9Cys, PLN-Arg14del and PLN-Arg25Cys, structural alterations are caused by these three variants (Hughes and Middleton, 2014; Liu et al., 2015; Vostrikov et al., 2015; Nelson et al., 2018; Vafiadaki et al., 2022). While their interaction to SERCA2 is impaired by all (Abrol et al., 2015; Liu et al., 2015; Vafiadaki et al., 2022), only PLN-Arg9Cys and PLN-Arg14del have been reported to exhibit additional aberrant interactions to proteins implicated in regulation of SERCA2 or PLN phosphorylation (Rigatti et al., 2015; Vafiadaki et al., 2022). In support of this, phosphorylation of PLN-Arg9Cys and PLN-Arg14del is abrogated while no change has been observed in the phosphorylation status of PLN-Arg25Cys (Schmitt et al., 2003; Ceholski et al., 2012b; Liu et al., 2015; Vafiadaki et al., 2022). These findings suggest that the presence of multiple, simultaneous alterations in critical PLN functional parameters may contribute towards development of a more severe spectrum of disease (i.e., DCM) in PLN-Arg9Cys and PLN-Arg14del, while a milder phenotype (i.e., HCM) is associated with PLN-Arg25Cys. It remains to be determined whether this differential topological effect on disease manifestation may be applicable to other *PLN* variants.

### 4.1 The pathogenic *PLN* variants involve predominantly loss of protein function

While the impact of published variants on PLN function has been characterized in only a small subset, it appears that the majority of pathogenic variants result in loss-of-function (Table 2). This observation, along with the fact that they are mainly found in heterozygous state, suggests a PLN dosage effect, with reduction in PLN function being central to disease pathogenesis as the presence of the wild-type PLN allele does not appear to suffice for normal cardiac function. A representative example is the particularly severe phenotype in the case of the dominant-negative variant Arg9Cys, with progressive heart failure requiring heart transplantation in early adult life, which has been described across unrelated families (Schmitt et al., 2003; Truszkowska et al., 2015; Fish et al., 2016). An exception to this loss-of-function effect appears to be the nonsense variant Glu2Ter, which has been observed in homozygosity in a 36 years old individual with severe DCM and heart failure, whereas heterozygous carriers were reported to have normal cardiac function (Li et al., 2019). It remains to be determined whether a single copy of this variant can lead to late onset disease or whether other mechanisms, such as incomplete penetrance, may be contributing to this apparent lack of cardiac phenotype. Another intriguing variant is Leu39Ter, which associates with a dosage-dependent effect correlating to disease severity. Heterozygotes exhibit HCM due to partial PLN functionality, while homozygotes develop severe DCM at a young age due to complete loss of PLN function (Landstrom et al., 2011).

**TABLE 2:** Functional effects and experimental evidence on currently characterized *PLN* variants.

*PLN* variant	Functional effect	Evidence type	Reference
c.40_42delAGA, p.Arg14del	Uncertain	*In vitro*, animal models, patient material	Haghighi et al. (2006), Karakikes et al. (2015), Eijgenraam et al. (2020), Badone et al. (2021), Cuello et al. (2021), Feyen et al. (2021), Grote Beverborg et al. (2021), Haghighi et al. (2021), Raad et al. (2021), Dave et al. (2022), Vafiadaki et al. (2022), Cleary et al. (2023), Rogalska et al. (2023), Stege et al. (2023), Cleary et al. (2024), Foo et al. (2024), Maniezzi et al. (2024), Vafiadaki et al. (2024)
c.25C>T, p.Arg9Cys	Loss of function	*In vitro*, animal models, patient material	Schmitt et al. (2003), Schmitt et al. (2009), Ha et al. (2011), Ceholski et al. (2012b), Abrol et al. (2015), Nelson et al. (2018), Vicente et al. (2022), Weber et al. (2024)
c.26G>T, p.Arg9Leu	Loss of function	*In vitro*	Medeiros et al. (2011), Ceholski et al. (2012a), Ceholski et al. (2012b), Weber et al. (2024)
c.26G>A, p.Arg9His	Loss of function	*In vitro*	Medeiros et al. (2011), Ceholski et al. (2012a), Weber et al. (2024)
c.43G>A, p.Ala15Thr	Gain of function	*In vitro*	Armanious et al. (2024)
c.53T>C; p.Ile18Thr	Partial loss of function	*In vitro*	Armanious et al. (2024)
c.61C>A, p.Pro21Thr	Gain of function	*In vitro*	Armanious et al. (2024)
c.73C>T, p.Arg25Cys	Gain of function	*In vitro*	Liu et al. (2015), Nelson et al. (2018)
c.4G>T, p.Glu2Ter	Loss of function	Patient material	Li et al. (2019)
c.116T>G, p.Leu39Ter	Loss of function	*In vitro*, patient material	Haghighi et al. (2003)

To date, the only reported gain-of-function variants comprise Ala15Thr, Pro21Thr and Arg25Cys, which have all been shown to result in increased SERCA inhibition. It is of interest that they are closely located along the length of the protein, with both Pro21Thr and Arg25Cys causing an increase in PLN helical structure that possibly indicates a common pathogenic mechanism (Liu et al., 2015; Armanious et al., 2024). PLN-Arg14del is another putative gain-of-function variant that was initially proposed to cause SERCA super-inhibition although subsequent studies challenged this observation (Haghighi et al., 2006; Badone et al., 2021; Maniezzi et al., 2024). Thus, the impact of PLN-Arg14del on SERCA activity remains to be clarified.

### 4.2 The role of genetic modifiers in *PLN* cardiomyopathies

The phenotype of carriers with pathogenic *PLN* variants is highly variable, with evidence of both incomplete penetrance and variable expressivity. Patients may present with DCM, HCM or ARVC, while the age of onset and the disease severity ranges from asymptomatic all the way to sudden cardiac death (SCD) or end-stage heart failure (HF) at a young age (Table 3). The phenotypic variability is evident even among carriers of the same *PLN* variant, such as PLN-Arg14del (Hof et al., 2019). Although the potential role of non-genetic parameters including exercise, chronic stress, personality traits, lifestyle factors and major disease manifestations has been assessed, no significant association has been established to date (van Drie et al., 2023; van Lint et al., 2023; Mahmoud et al., 2025). Meanwhile, the implication of genetic modifiers has long been suspected, and evidence to support this is being explored.

**TABLE 3:** *PLN* variants, clinical characteristics and cases reported to date.

Type of variant	*PLN* variant	Domain location	Clinical phenotype	Age of onset	Penetrance	Number of reported cases	Reference
Deletion	c.40_42delAGA, p.Arg14del	Cytoplasmic	Asymptomatic, ACM or DCM with arrhythmias	30–60 years, arrhythmias may occur at earlier age	Incomplete	>1,500	Haghighi et al. (2003), van der Zwaag et al. (2012), van Rijsingen et al. (2014), Cheung et al. (2019), Hof et al. (2019), Jiang et al. (2020), Tabata et al. (2021), Verstraelen et al. (2025)
	c.95_98delTTAT, p.Phe32SerfsTer7	Cytoplasmic	HCM	14 years	Unknown	1	Du et al. (2024)
Missense	c.23C>T, p.Thr8Ile	Cytoplasmic	DCM	Unknown	Unknown	Not specified	Sousa et al. (2019)
	c.25C>T, p.Arg9Cys	Cytoplasmic	DCM	20–35 years	Complete	10	Schmitt et al. (2003), Truszkowska et al. (2015), Fish et al. (2016)
	c.26G>T, p.Arg9Leu	Cytoplasmic	DCM	30–50 years	Unknown	4	Medeiros et al. (2011), Truszkowska et al. (2015)
	c.26G>A, p.Arg9His	Cytoplasmic	DCM	43 years	Incomplete	>7 (Not specified in Sousa et al.)	Medeiros et al. (2011), Sousa et al. (2019)
	c.43G>A, p.Ala15Thr	Cytoplasmic	DCM	4 years	Unknown	1	Pugh et al. (2014)
	c.46T>G, p.Ser16Pro	Cytoplasmic	HCM	Unknown	Unknown	1	Walsh et al. (2017b)
	c.50C>A, pThr17Asn	Cytoplasmic	DCM	44 years	Unknown	1	Mollanoori et al. (2018)
	c.53T>C; p.Ile18Thr	Cytoplasmic	HCM	Unknown	Unknown	>1 (Not specified in Lopes et al.)	Burns et al. (2017), Lopes et al. (2015)
	c.61C>A, p.Pro21Thr	Cytoplasmic	DCM	60 years	Unknown	>1 (Not specified in Sousa et al.)	Pugh et al. (2014), Sousa et al. (2019)
	c.73C>T, p.Arg25Cys	Cytoplasmic	DCM, HCM and Bruganda syndrome	45 years		>5 (Not specified in Lopes et al.)	Behr et al. (2015), Liu et al. (2015), Lopes et al. (2015)
	c.121T>A, p.Cys41Ser	Transmembrane	HCM	Unknown	Unknown	1	Walsh et al. (2017b)
	c.145G>A, p.Val49Met	Transmembrane	HCM	Unknown	Unknown	1	Xu et al. (2015)
	c.152T>C, p.Leu51Pro	Transmembrane	HCM	Unknown	Unknown	>1 (Not specified in Lopes et al.)	Walsh et al. (2017b), Lopes et al. (2015)
Nonsense	c.4G>T, p.Glu2Ter	Cytoplasmic	DCM and severe heart failure, arrhythmias	36 years	Unknown	6	Li et al. (2019)
	c.116T>G, p.Leu39Ter	Transmembrane	Asymptomatic hypertrophy, HCM or severe DCM with cardiac arrhythmias	17–51 years	Incomplete	28	Haghighi et al. (2003), Landstrom et al. (2011), Medeiros et al. (2011), Sanoudou et al. (2015), Walsh et al. (2017a), Walsh et al. (2017b), Mazzone et al. (2024), Burns et al. (2017)
Insertion	c.9_10insA, p.Val4SerFsTer15	Cytoplasmic	Wolf-Parkinson-White syndrome with unconfirmed diagnosis of cardiomyopathy	31 years	Unknown	1	Truszkowska et al. (2015)
	c.37_38insA, p.Arg13LysfsTer7	Cytoplasmic	DCM	39 years	Family history of DCM	1	Pugh et al. (2014)
	c.61_62insCT, p.Pro21LeufsTer19	Cytoplasmic	HCM	Unknown	Unknown	Not specified	Lopes et al. (2015)
	c.63_64dupTC, p.Gln22LeufsTer19	Cytoplasmic	HCM	Unknown	Unknown	1	Walsh et al. (2017b)
	c.138dupT, p.Ile47TyrfsTer14	Transmembrane	HCM	Unknown	Unknown	1	Walsh et al. (2017b)
Promoter	−77 A>G	Promoter	HCM	56 years	Unknown	1	Minamisawa et al. (2003)
	−42 C>G	Promoter	HCM and healthy	67 years	Incomplete	3	Medin et al. (2007)
	−36 A>C	Promoter	DCM and healthy	18–44 years	Incomplete	24	Medin et al. (2007), Haghighi et al. (2008)

A tentative effect of individual modifier genes has also been proposed in relation to different *PLN* variants. For example, although PLN-Leu39Ter heterozygosity has been reported to lead to HCM, in the presence of three additional genetic variants in DCM/arrhythmia associated genes, a PLN-Leu39Ter heterozygote patient presented with DCM and sustained ventricular tachycardia (Sanoudou et al., 2015). Similarly, PLN-Arg25Cys has been identified in patients presenting with HCM or Brugada. However in the presence of both PLN-Arg25Cys and a *LMNA* variant severe DCM was observed, with the patient requiring heart transplantation (Behr et al., 2015; Liu et al., 2015; Lopes et al., 2015).

These observations in *PLN* cardiomyopathy patients are well-aligned with increasing evidence supporting the coexistence of multiple genetic variants in a proportion of DCM patients (Haas et al., 2015; Pankuweit and Richter, 2015). In specific, it was recently proposed that the overall genetic landscape underlying DCM and HCM is significantly different, with increased coexistence of genetic variants correlating with disease severity and predisposition to DCM, compared to HCM (Puckelwartz et al., 2021). For example, *BAG3* Cys151Arg was proposed to serve as an important genetic modifier variant in DCM, by modulating risk on the DCM-HCM spectrum, and impacting DCM risk in carriers of pathogenic truncating titin variants (Park et al., 2024). Similarly, a variant of unknown significance in the myosin heavy chain 7 gene (*MYH7* p. Ile1927Phe) was shown to contribute to the development of severe HCM in the presence of truncating *MyBPC3* variants (Escriba et al., 2023).

## 5 Conclusion and perspective

PLN has a well-established and central role in cardiac function and disease. Pathogenic *PLN* variants, span missense, nonsense, small deletions, and insertions that are associated with a range of cardiomyopathy phenotypes. Although only some of these variants have been functionally characterized, their analysis has contributed to elucidation of key pathogenic mechanisms, such as altered Ca^2+^ handling, structural disruption, and aberrant protein interactions. In public genetic databases many more novel *PLN* variants have been submitted, including predicted P/LP, even though experimental validation is pending. The increasing use of genetic testing as part of the Cardiology Clinics’ routine is anticipated to unveil a greater number and frequency of *PLN* variants. Importantly, pathogenic *PLN* variants are associated with a spectrum of cardiomyopathy-related phenotypes. Detailed mapping of these variants, careful characterization of their biological role, and investigation of genotype-phenotype correlations, has the potential to transform healthcare for patients with *PLN* variants, by enhancing predictive, diagnostic, and prognostic accuracy, as well as the precision of future therapeutic approaches. Towards this, the development of targeted interventions, guided by the unique properties of specific *PLN* variants, represents a promising avenue to address the unmet clinical needs of the patients and ultimately mitigate PLN-related cardiac diseases. For Cardiology clinics to reap the full potential of these fascinating developments, close interaction with genetics experts and genetic counselors is recommended (Musunuru et al., 2020; Wilde et al., 2022; Morales et al., 2023; Goehringer et al., 2025; Sanoudou et al., 2025). Meanwhile, as current understanding of genetic, epigenetic and epitranscriptomic variation evolves, and the artificial intelligence milieu matures, more sophisticated all-encompassing clinical prediction/classification/prognostic/prevention tools are foreseen (Kalozoumi et al., 2012; Leptidis et al., 2022; Sel et al., 2024).

## References

[B1] Abrol N. de Tombe P. P. Robia S. L. (2015). Acute inotropic and lusitropic effects of cardiomyopathic R9C mutation of phospholamban. J. Biol. Chem. 290 (11), 7130–7140. 10.1074/jbc.M114.630319 25593317 PMC4358133

[B2] Abrol N. Smolin N. Armanious G. Ceholski D. K. Trieber C. A. Young H. S. (2014). Phospholamban C-terminal residues are critical determinants of the structure and function of the calcium ATPase regulatory complex. J. Biol. Chem. 289 (37), 25855–25866. 10.1074/jbc.M114.562579 25074938 PMC4162186

[B3] Alfares A. A. Kelly M. A. McDermott G. Funke B. H. Lebo M. S. Baxter S. B. (2015). Results of clinical genetic testing of 2,912 probands with hypertrophic cardiomyopathy: expanded panels offer limited additional sensitivity. Genet. Med. 17 (11), 880–888. 10.1038/gim.2014.205 25611685

[B4] Arbelo E. Protonotarios A. Gimeno J. R. Arbustini E. Barriales-Villa R. Basso C. (2023). 2023 ESC Guidelines for the management of cardiomyopathies. Eur. Heart J. 44 (37), 3503–3626. 10.1093/eurheartj/ehad194 37622657

[B5] Armanious G. P. Lemieux M. J. Espinoza-Fonseca L. M. Young H. S. (2024). Missense variants in phospholamban and cardiac myosin binding protein identified in patients with a family history and clinical diagnosis of dilated cardiomyopathy. Biochim. Biophys. Acta Mol. Cell Res. 1871 (4), 119699. 10.1016/j.bbamcr.2024.119699 38387507

[B6] Asahi M. Kimura Y. Kurzydlowski K. Tada M. MacLennan D. H. (1999). Transmembrane helix M6 in sarco(endo)plasmic reticulum Ca(2+)-ATPase forms a functional interaction site with phospholamban. Evidence for physical interactions at other sites. J. Biol. Chem. 274 (46), 32855–32862. 10.1074/jbc.274.46.32855 10551848

[B7] Asahi M. Nakayama H. Tada M. Otsu K. (2003). Regulation of sarco(endo)plasmic reticulum Ca2+ adenosine triphosphatase by phospholamban and sarcolipin: implication for cardiac hypertrophy and failure. Trends Cardiovasc Med. 13 (4), 152–157. 10.1016/s1050-1738(03)00037-9 12732449

[B8] Badone B. Ronchi C. Lodola F. Knaust A. E. Hansen A. Eschenhagen T. (2021). Characterization of the PLN p.Arg14del Mutation in Human Induced Pluripotent Stem Cell-Derived Cardiomyocytes. Int. J. Mol. Sci. 22 (24), 13500. 10.3390/ijms222413500 34948294 PMC8709382

[B9] Behr E. R. Savio-Galimberti E. Barc J. Holst A. G. Petropoulou E. Prins B. P. (2015). Role of common and rare variants in SCN10A: results from the Brugada syndrome QRS locus gene discovery collaborative study. Cardiovasc Res. 106 (3), 520–529. 10.1093/cvr/cvv042 25691538 PMC4447806

[B10] Belakavadi M. Saunders J. Weisleder N. Raghava P. S. Fondell J. D. (2010). Repression of cardiac phospholamban gene expression is mediated by thyroid hormone receptor-{alpha}1 and involves targeted covalent histone modifications. Endocrinology 151 (6), 2946–2956. 10.1210/en.2009-1241 20392835 PMC2875831

[B11] Bers D. M. (2002). Cardiac excitation-contraction coupling. Nature 415 (6868), 198–205. 10.1038/415198a 11805843

[B12] Bleijendaal H. Ramos L. A. Lopes R. R. Verstraelen T. E. Baalman S. W. E. Oudkerk Pool M. D. (2021). Computer versus cardiologist: Is a machine learning algorithm able to outperform an expert in diagnosing a phospholamban p.Arg14del mutation on the electrocardiogram? Heart rhythm. 18 (1), 79–87. 10.1016/j.hrthm.2020.08.021 32911053

[B13] Burns C. Bagnall R. D. Lam L. Semsarian C. Ingles J. (2017). Multiple gene variants in hypertrophic cardiomyopathy in the era of next-generation sequencing. Circ. Cardiovasc Genet. 10 (4), e001666. 10.1161/CIRCGENETICS.116.001666 28790153

[B14] Ceholski D. K. Trieber C. A. Holmes C. F. Young H. S. (2012a). Lethal, hereditary mutants of phospholamban elude phosphorylation by protein kinase A. J. Biol. Chem. 287 (32), 26596–26605. 10.1074/jbc.M112.382713 22707725 PMC3411000

[B15] Ceholski D. K. Trieber C. A. Young H. S. (2012b). Hydrophobic imbalance in the cytoplasmic domain of phospholamban is a determinant for lethal dilated cardiomyopathy. J. Biol. Chem. 287 (20), 16521–16529. 10.1074/jbc.M112.360859 22427649 PMC3351288

[B16] Ceholski D. K. Turnbull I. C. Kong C. W. Koplev S. Mayourian J. Gorski P. A. (2018). Functional and transcriptomic insights into pathogenesis of R9C phospholamban mutation using human induced pluripotent stem cell-derived cardiomyocytes. J. Mol. Cell Cardiol. 119, 147–154. 10.1016/j.yjmcc.2018.05.007 29752948 PMC6039110

[B17] Cheung C. C. Healey J. S. Hamilton R. Spears D. Gollob M. H. Mellor G. (2019). Phospholamban cardiomyopathy: a Canadian perspective on a unique population. Neth Heart J. 27 (4), 208–213. 10.1007/s12471-019-1247-0 30806910 PMC6439019

[B18] Chiu C. Tebo M. Ingles J. Yeates L. Arthur J. W. Lind J. M. (2007). Genetic screening of calcium regulation genes in familial hypertrophic cardiomyopathy. J. Mol. Cell Cardiol. 43 (3), 337–343. 10.1016/j.yjmcc.2007.06.009 17655857

[B19] Cleary S. R. Seflova J. Cho E. E. Bisht K. Khandelia H. Espinoza-Fonseca L. M. (2024). Phospholamban inhibits the cardiac calcium pump by interrupting an allosteric activation pathway. J. Biol. Chem. 300 (5), 107267. 10.1016/j.jbc.2024.107267 38583863 PMC11098958

[B20] Cleary S. R. Teng A. C. T. Kongmeneck A. D. Fang X. Phillips T. A. Cho E. E. (2023). Dilated cardiomyopathy variant R14del increases phospholamban pentamer stability, blunting dynamic regulation of cardiac calcium handling. bioRxiv. 10.1101/2023.05.26.542463 PMC11791128 39710323

[B21] Cuello F. Knaust A. E. Saleem U. Loos M. Raabe J. Mosqueira D. (2021). Impairment of the ER/mitochondria compartment in human cardiomyocytes with PLN p.Arg14del mutation. EMBO Mol. Med. 13 (6), e13074. 10.15252/emmm.202013074 33998164 PMC8185541

[B22] Dave J. Raad N. Mittal N. Zhang L. Fargnoli A. Oh J. G. (2022). Gene editing reverses arrhythmia susceptibility in humanized PLN-R14del mice: modelling a European cardiomyopathy with global impact. Cardiovasc Res. 118 (15), 3140–3150. 10.1093/cvr/cvac021 35191471 PMC9732517

[B133] Deiman F. E. Bomer N. van der Meer P. Grote Beverborg N. (2022). Review: precision medicine approaches for genetic cardiomyopathy: targeting phospholamban R14del. Curr. Heart Fail. Rep. 10.1007/s11897-022-00558-x PMC9329159 35699837

[B23] Doevendans P. A. Glijnis P. C. Kranias E. G. (2019). Leducq transatlantic network of excellence to cure phospholamban-induced cardiomyopathy (CURE-PLaN). Circ. Res. 125 (7), 720–724. 10.1161/CIRCRESAHA.119.315077 31513489

[B24] Du X. Barnett C. L. Widmeyer K. M. Wang X. Brightman D. S. Noonan C. W. (2024). RMND1 and PLN variants are the underlying cause of Perrault-like syndrome and cardiac anomalies in a patient. Clin. Case Rep. 12 (11), e9537. 10.1002/ccr3.9537 39493792 PMC11527736

[B25] Eijgenraam T. R. Boukens B. J. Boogerd C. J. Schouten E. M. van de Kolk C. W. A. Stege N. M. (2020). The phospholamban p.(Arg14del) pathogenic variant leads to cardiomyopathy with heart failure and is unreponsive to standard heart failure therapy. Sci. Rep. 10 (1), 9819. 10.1038/s41598-020-66656-9 32555305 PMC7300032

[B26] Eijgenraam T. R. Stege N. M. Oliveira Nunes Teixeira V. de Brouwer R. Schouten E. M. Grote Beverborg N. (2022). Antisense Therapy Attenuates Phospholamban p.(Arg14del) Cardiomyopathy in Mice and Reverses Protein Aggregation. Int. J. Mol. Sci. 23 (5), 2427. 10.3390/ijms23052427 35269571 PMC8909937

[B27] Escriba R. Larranaga-Moreira J. M. Richaud-Patin Y. Pourchet L. Lazis I. Jimenez-Delgado S. (2023). iPSC-based modeling of variable clinical presentation in hypertrophic cardiomyopathy. Circ. Res. 133 (2), 108–119. 10.1161/CIRCRESAHA.122.321951 37317833

[B28] Feyen D. A. M. Perea-Gil I. Maas R. G. C. Harakalova M. Gavidia A. A. Arthur Ataam J. (2021). Unfolded Protein Response as a Compensatory Mechanism and Potential Therapeutic Target in PLN R14del Cardiomyopathy. Circulation 144 (5), 382–392. 10.1161/CIRCULATIONAHA.120.049844 33928785 PMC8667423

[B29] Fish M. Shaboodien G. Kraus S. Sliwa K. Seidman C. E. Burke M. A. (2016). Mutation analysis of the phospholamban gene in 315 South Africans with dilated, hypertrophic, peripartum and arrhythmogenic right ventricular cardiomyopathies. Sci. Rep. 6, 22235. 10.1038/srep22235 26917049 PMC4808831

[B30] Foo B. Amedei H. Kaur S. Jaawan S. Boshnakovska A. Gall T. (2024). Unbiased complexome profiling and global proteomics analysis reveals mitochondrial impairment and potential changes at the intercalated disk in presymptomatic R14Δ/+ mice hearts. PLoS One 19 (10), e0311203. 10.1371/journal.pone.0311203 39446877 PMC11501035

[B31] Froehlich J. P. Mahaney J. E. Keceli G. Pavlos C. M. Goldstein R. Redwood A. J. (2008). Phospholamban thiols play a central role in activation of the cardiac muscle sarcoplasmic reticulum calcium pump by nitroxyl. Biochemistry 47 (50), 13150–13152. 10.1021/bi801925p 19053265

[B32] Fujii J. Zarain-Herzberg A. Willard H. F. Tada M. MacLennan D. H. (1991). Structure of the rabbit phospholamban gene, cloning of the human cDNA, and assignment of the gene to human chromosome 6. J. Biol. Chem. 266 (18), 11669–11675. 10.1016/s0021-9258(18)99009-5 1828805

[B33] Ganim J. R. Luo W. Ponniah S. Grupp I. Kim H. W. Ferguson D. G. (1992). Mouse phospholamban gene expression during development *in vivo* and *in vitro* . Circ. Res. 71 (5), 1021–1030. 10.1161/01.res.71.5.1021 1394867

[B34] Goehringer J. Sanoudou D. Morales A. (2025). “Genetic counseling for cardiovascular disease: Part A – pre-test approaches and considerations,” in Genetic counselling - navigating the future. Editor Seifi M. (London, United Kingdom: IntechOpen). 10.5772/intechopen.1007908

[B35] Grote Beverborg N. Spater D. Knoll R. Hidalgo A. Yeh S. T. Elbeck Z. (2021). Phospholamban antisense oligonucleotides improve cardiac function in murine cardiomyopathy. Nat. Commun. 12 (1), 5180. 10.1038/s41467-021-25439-0 34462437 PMC8405807

[B36] Gustavsson M. Traaseth N. J. Karim C. B. Lockamy E. L. Thomas D. D. Veglia G. (2011). Lipid-mediated folding/unfolding of phospholamban as a regulatory mechanism for the sarcoplasmic reticulum Ca2+-ATPase. J. Mol. Biol. 408 (4), 755–765. 10.1016/j.jmb.2011.03.015 21419777 PMC3573877

[B37] Gustavsson M. Verardi R. Mullen D. G. Mote K. R. Traaseth N. J. Gopinath T. (2013). Allosteric regulation of SERCA by phosphorylation-mediated conformational shift of phospholamban. Proc. Natl. Acad. Sci. U. S. A. 110 (43), 17338–17343. 10.1073/pnas.1303006110 24101520 PMC3808617

[B38] Ha K. N. Masterson L. R. Hou Z. Verardi R. Walsh N. Veglia G. (2011). Lethal Arg9Cys phospholamban mutation hinders Ca2+-ATPase regulation and phosphorylation by protein kinase A. Proc. Natl. Acad. Sci. U. S. A. 108 (7), 2735–2740. 10.1073/pnas.1013987108 21282613 PMC3041113

[B39] Haas J. Frese K. S. Peil B. Kloos W. Keller A. Nietsch R. (2015). Atlas of the clinical genetics of human dilated cardiomyopathy. Eur. Heart J. 36 (18), 1123–135a. 10.1093/eurheartj/ehu301 25163546

[B40] Haghighi K. Bidwell P. Kranias E. G. (2014). Phospholamban interactome in cardiac contractility and survival: a new vision of an old friend. J. Mol. Cell Cardiol. 77, 160–167. 10.1016/j.yjmcc.2014.10.005 25451386 PMC4312245

[B41] Haghighi K. Chen G. Sato Y. Fan G. C. He S. Kolokathis F. (2008). A human phospholamban promoter polymorphism in dilated cardiomyopathy alters transcriptional regulation by glucocorticoids. Hum. Mutat. 29 (5), 640–647. 10.1002/humu.20692 18241046 PMC5074532

[B42] Haghighi K. Gardner G. Vafiadaki E. Kumar M. Green L. C. Ma J. (2021). Impaired right ventricular calcium cycling is an early risk factor in r14del-phospholamban arrhythmias. J. Pers. Med. 11 (6), 502. 10.3390/jpm11060502 34204946 PMC8226909

[B43] Haghighi K. Kolokathis F. Gramolini A. O. Waggoner J. R. Pater L. Lynch R. A. (2006). A mutation in the human phospholamban gene, deleting arginine 14, results in lethal, hereditary cardiomyopathy. Proc. Natl. Acad. Sci. U. S. A. 103 (5), 1388–1393. 10.1073/pnas.0510519103 16432188 PMC1360586

[B44] Haghighi K. Kolokathis F. Pater L. Lynch R. A. Asahi M. Gramolini A. O. (2003). Human phospholamban null results in lethal dilated cardiomyopathy revealing a critical difference between mouse and human. J. Clin. Invest 111 (6), 869–876. 10.1172/JCI17892 12639993 PMC153772

[B45] Hirtle-Lewis M. Desbiens K. Ruel I. Rudzicz N. Genest J. Engert J. C. (2013). The genetics of dilated cardiomyopathy: a prioritized candidate gene study of LMNA, TNNT2, TCAP, and PLN. Clin. Cardiol. 36 (10), 628–633. 10.1002/clc.22193 24037902 PMC6649360

[B46] Hof I. E. van der Heijden J. F. Kranias E. G. Sanoudou D. de Boer R. A. van Tintelen J. P. (2019). Prevalence and cardiac phenotype of patients with a phospholamban mutation. Neth Heart J. 27 (2), 64–69. 10.1007/s12471-018-1211-4 30547415 PMC6352623

[B47] Hu H. Jiang M. Cao Y. Zhang Z. Jiang B. Tian F. (2020). HuR regulates phospholamban expression in isoproterenol-induced cardiac remodelling. Cardiovasc Res. 116 (5), 944–955. 10.1093/cvr/cvz205 31373621 PMC7868665

[B48] Hughes E. Middleton D. A. (2014). Comparison of the structure and function of phospholamban and the arginine-14 deficient mutant associated with dilated cardiomyopathy. PLoS One 9 (9), e106746. 10.1371/journal.pone.0106746 25225809 PMC4165587

[B49] Irie T. Sips P. Y. Kai S. Kida K. Ikeda K. Hirai S. (2015). S-nitrosylation of calcium-handling proteins in cardiac adrenergic signaling and hypertrophy. Circ. Res. 117 (9), 793–803. 10.1161/CIRCRESAHA.115.307157 26259881 PMC4600453

[B50] Jiang X. Xu Y. Sun J. Wang L. Guo X. Chen Y. (2020). The phenotypic characteristic observed by cardiac magnetic resonance in a PLN-R14del family. Sci. Rep. 10 (1), 16478. 10.1038/s41598-020-73359-8 33020536 PMC7536202

[B51] Kalozoumi G. Tzimas C. Sanoudou D. (2012). The expanding role of epigenetics. Glob. Cardiol. Sci. Pract. 2012 (1), 7. 10.5339/gcsp.2012.7 25610838 PMC4239821

[B52] Karakikes I. Stillitano F. Nonnenmacher M. Tzimas C. Sanoudou D. Termglinchan V. (2015). Correction of human phospholamban R14del mutation associated with cardiomyopathy using targeted nucleases and combination therapy. Nat. Commun. 6, 6955. 10.1038/ncomms7955 25923014 PMC4421839

[B53] Keceli G. Majumdar A. Thorpe C. N. Jun S. Tocchetti C. G. Lee D. I. (2019). Nitroxyl (HNO) targets phospholamban cysteines 41 and 46 to enhance cardiac function. J. Gen. Physiol. 151 (6), 758–770. 10.1085/jgp.201812208 30842219 PMC6571998

[B54] Kimura Y. Asahi M. Kurzydlowski K. Tada M. MacLennan D. H. (1998). Phospholamban domain Ib mutations influence functional interactions with the Ca2+-ATPase isoform of cardiac sarcoplasmic reticulum. J. Biol. Chem. 273 (23), 14238–14241. 10.1074/jbc.273.23.14238 9603928

[B55] Kimura Y. Kurzydlowski K. Tada M. MacLennan D. H. (1996). Phospholamban regulates the Ca2+-ATPase through intramembrane interactions. J. Biol. Chem. 271 (36), 21726–21731. 10.1074/jbc.271.36.21726 8702967

[B56] Kranias E. G. Doevendans P. A. Glijnis P. C. Hajjar R. J. (2018). PLN foundation. Circulation Res. 123 (12), 1276–1278. 10.1161/Circresaha.118.314014 30566043 PMC6363126

[B57] Kranias E. G. Hajjar R. J. (2017). The phospholamban journey 4 decades after setting out for ithaka. Circ. Res. 120 (5), 781–783. 10.1161/CIRCRESAHA.116.310007 28254803 PMC5338645

[B58] Landstrom A. P. Adekola B. A. Bos J. M. Ommen S. R. Ackerman M. J. (2011). PLN-encoded phospholamban mutation in a large cohort of hypertrophic cardiomyopathy cases: summary of the literature and implications for genetic testing. Am. Heart J. 161 (1), 165–171. 10.1016/j.ahj.2010.08.001 21167350 PMC6311091

[B59] Leptidis S. Papakonstantinou E. Diakou K. I. Pierouli K. Mitsis T. Dragoumani K. (2022). Epitranscriptomics of cardiovascular diseases (Review). Int. J. Mol. Med. 49 (1), 9. (Review). 10.3892/ijmm.2021.5064 34791505 PMC8651226

[B60] Li Z. Chen P. Xu J. Yu B. Li X. Wang D. W. (2019). A PLN nonsense variant causes severe dilated cardiomyopathy in a novel autosomal recessive inheritance mode. Int. J. Cardiol. 279, 122–125. 10.1016/j.ijcard.2018.12.075 30638982

[B61] Liang X. H. Sun H. Shen W. Wang S. Yao J. Migawa M. T. (2017). Antisense oligonucleotides targeting translation inhibitory elements in 5' UTRs can selectively increase protein levels. Nucleic Acids Res. 45 (16), 9528–9546. 10.1093/nar/gkx632 28934489 PMC5766168

[B62] Liu G. S. Morales A. Vafiadaki E. Lam C. K. Cai W. F. Haghighi K. (2015). A novel human R25C-phospholamban mutation is associated with super-inhibition of calcium cycling and ventricular arrhythmia. Cardiovasc Res. 107 (1), 164–174. 10.1093/cvr/cvv127 25852082 PMC4490203

[B63] Lopes L. R. Syrris P. Guttmann O. P. O'Mahony C. Tang H. C. Dalageorgou C. (2015). Novel genotype-phenotype associations demonstrated by high-throughput sequencing in patients with hypertrophic cardiomyopathy. Heart 101 (4), 294–301. 10.1136/heartjnl-2014-306387 25351510 PMC4345808

[B64] Lopes R. R. Bleijendaal H. Ramos L. A. Verstraelen T. E. Amin A. S. Wilde A. A. M. (2021). Improving electrocardiogram-based detection of rare genetic heart disease using transfer learning: An application to phospholamban p.Arg14del mutation carriers. Comput. Biol. Med. 131, 104262. 10.1016/j.compbiomed.2021.104262 33607378

[B65] MacLennan D. H. Kimura Y. Toyofuku T. (1998). Sites of regulatory interaction between calcium ATPases and phospholamban. Ann. N. Y. Acad. Sci. 853, 31–42. 10.1111/j.1749-6632.1998.tb08254.x 10603934

[B66] MacLennan D. H. Kranias E. G. (2003). Phospholamban: a crucial regulator of cardiac contractility. Nat. Rev. Mol. Cell Biol. 4 (7), 566–577. 10.1038/nrm1151 12838339

[B67] Mahmoud B. van der Heide M. Y. C. Cox M. Verstraelen T. E. de Brouwer R. van Drie E. (2025). The role of Comorbidities and lifestyle factors in disease progression of phospholamban cardiomyopathy. Eur. J. Prev. Cardiol., zwaf084. 10.1093/eurjpc/zwaf084 39969823

[B68] Maniezzi C. Eskandr M. Florindi C. Ferrandi M. Barassi P. Sacco E. (2024). Early consequences of the phospholamban mutation PLN-R14del(+/-) in a transgenic mouse model. Acta Physiol. (Oxf) 240 (3), e14082. 10.1111/apha.14082 38214033

[B69] Mattiazzi A. Kranias E. G. (2024). Unleashing the Power of genetics: PLN Ablation, Phospholambanopathies and evolving challenges. Circ. Res. 134 (2), 138–142. 10.1161/CIRCRESAHA.123.323053 38236951

[B70] Mayr C. (2019). What are 3' UTRs doing? Cold Spring Harb. Perspect. Biol. 11 (10), a034728. 10.1101/cshperspect.a034728 30181377 PMC6771366

[B71] Mazzone F. Giovannico L. Fischetti G. Parigino D. Guaricci A. I. Forleo C. (2024). Homozygous phospholamban mutation causing dilated cardiomyopathy in a young man: from cardiogenic Shock to Tennis Tournaments. Clin. Transpl. 38 (11), e70031. 10.1111/ctr.70031 39585194

[B72] Medeiros A. Biagi D. G. Sobreira T. J. de Oliveira P. S. Negrao C. E. Mansur A. J. (2011). Mutations in the human phospholamban gene in patients with heart failure. Am. Heart J. 162 (6), 1088–1095. 10.1016/j.ahj.2011.07.028 22137083

[B73] Medin M. Hermida-Prieto M. Monserrat L. Laredo R. Rodriguez-Rey J. C. Fernandez X. (2007). Mutational screening of phospholamban gene in hypertrophic and idiopathic dilated cardiomyopathy and functional study of the PLN -42 C>G mutation. Eur. J. Heart Fail 9 (1), 37–43. 10.1016/j.ejheart.2006.04.007 16829191

[B74] Minamisawa S. Sato Y. Tatsuguchi Y. Fujino T. Imamura S. Uetsuka Y. (2003). Mutation of the phospholamban promoter associated with hypertrophic cardiomyopathy. Biochem. Biophys. Res. Commun. 304 (1), 1–4. 10.1016/s0006-291x(03)00526-6 12705874

[B75] Mollanoori H. Naderi N. Amin A. Hassani B. Shahraki H. Teimourian s. (2018). A novel human T17N-phospholamban variation in idiopathic dilated cardiomyopathy. Gene Rep. 12, 122–127. 10.1016/j.genrep.2018.06.014

[B76] Morales A. Goehringer J. Sanoudou D. (2023). Evolving cardiovascular genetic counseling needs in the era of precision medicine. Front. Cardiovasc Med. 10, 1161029. 10.3389/fcvm.2023.1161029 37424912 PMC10325680

[B77] Mushala B. Cho E. Stoner M. Gibson G. Sembrat J. Bugga P. (2024). Abstract Mo069: phospholamban acetylation Enhances cardiomyocyte calcium cycling under conditions of high-Fat Feeding. Circulation Res. 135 (Suppl. 1), AMo069. 10.1161/res.135.suppl_1.Mo069

[B78] Musunuru K. Hershberger R. E. Day S. M. Klinedinst N. J. Landstrom A. P. Parikh V. N. (2020). Genetic testing for inherited cardiovascular diseases: a Scientific statement from the American heart association. Circ. Genom Precis. Med. 13 (4), e000067. 10.1161/HCG.0000000000000067 32698598

[B79] Nakagawa T. Yokoe S. Asahi M. (2016). Phospholamban degradation is induced by phosphorylation-mediated ubiquitination and inhibited by interaction with cardiac type Sarco(endo)plasmic reticulum Ca(2+)-ATPase. Biochem. Biophys. Res. Commun. 472 (3), 523–530. 10.1016/j.bbrc.2016.03.009 26966065

[B80] Nelson S. E. D. Ha K. N. Gopinath T. Exline M. H. Mascioni A. Thomas D. D. (2018). Effects of the Arg9Cys and Arg25Cys mutations on phospholamban's conformational equilibrium in membrane bilayers. Biochim. Biophys. Acta Biomembr. 1860 (6), 1335–1341. 10.1016/j.bbamem.2018.02.030 29501609 PMC6428084

[B81] Pankuweit S. Richter A. (2015). Clinical genetics of dilated cardiomyopathy: on the way to personalized medicine? Eur. Heart J. 36 (18), 1074–1077. 10.1093/eurheartj/ehu402 25336217

[B82] Park J. Levin M. G. Zhang D. Reza N. Mead J. O. Carruth E. D. (2024). Bidirectional risk modulator and modifier variant of dilated and hypertrophic cardiomyopathy in BAG3. JAMA Cardiol. 9 (12), 1124–1133. 10.1001/jamacardio.2024.3547 39535783 PMC11561727

[B83] Puckelwartz M. J. Pesce L. L. Dellefave-Castillo L. M. Wheeler M. T. Pottinger T. D. Robinson A. C. (2021). Genomic Context Differs between human dilated cardiomyopathy and hypertrophic cardiomyopathy. J. Am. Heart Assoc. 10 (7), e019944. 10.1161/JAHA.120.019944 33764162 PMC8174318

[B84] Pugh T. J. Kelly M. A. Gowrisankar S. Hynes E. Seidman M. A. Baxter S. M. (2014). The landscape of genetic variation in dilated cardiomyopathy as surveyed by clinical DNA sequencing. Genet. Med. 16 (8), 601–608. 10.1038/gim.2013.204 24503780

[B85] Raad N. Bittihn P. Cacheux M. Jeong D. Ilkan Z. Ceholski D. (2021). Arrhythmia Mechanism and Dynamics in a Humanized Mouse Model of Inherited Cardiomyopathy Caused by Phospholamban R14del Mutation. Circulation 144 (6), 441–454. 10.1161/CIRCULATIONAHA.119.043502 34024116 PMC8456417

[B86] Raguimova O. N. Aguayo-Ortiz R. Robia S. L. Espinoza-Fonseca L. M. (2020). Dynamics-driven Allostery underlies Ca(2+)-mediated release of SERCA inhibition by phospholamban. Biophys. J. 119 (9), 1917–1926. 10.1016/j.bpj.2020.09.014 33069270 PMC7677127

[B87] Ren A. J. Wei C. Liu Y. J. Liu M. Wang P. Fan J. (2024). ZBTB20 regulates SERCA2a activity and Myocardial contractility through phospholamban. Circ. Res. 134 (3), 252–265. 10.1161/CIRCRESAHA.123.323798 38166470

[B88] Rigatti M. Le A. V. Gerber C. Moraru I. I. Dodge-Kafka K. L. (2015). Phosphorylation state-dependent interaction between AKAP7δ/γ and phospholamban increases phospholamban phosphorylation. Cell Signal 27 (9), 1807–1815. 10.1016/j.cellsig.2015.05.016 26027516 PMC4787601

[B89] Roberts T. C. Langer R. Wood M. J. A. (2020). Advances in oligonucleotide drug delivery. Nat. Rev. Drug Discov. 19 (10), 673–694. 10.1038/s41573-020-0075-7 32782413 PMC7419031

[B90] Rogalska M. Vafiadaki E. Erpapazoglou Z. Haghighi K. Green L. Mantzoros C. S. (2023). Isoform changes of action potential regulators in the ventricles of arrhythmogenic phospholamban-R14del humanized mouse hearts. Metabolism 138, 155344. 10.1016/j.metabol.2022.155344 36375644 PMC12758590

[B91] Rogers H. T. Roberts D. S. Larson E. J. Melby J. A. Rossler K. J. Carr A. V. (2023). Comprehensive characterization of Endogenous phospholamban Proteoforms enabled by Photocleavable Surfactant and Top-down proteomics. Anal. Chem. 95 (35), 13091–13100. 10.1021/acs.analchem.3c01618 37607050 PMC10597709

[B92] Rohner E. Witman N. Sohlmer J. De Genst E. Louch W. E. Sahara M. (2021). An mRNA assay system demonstrates proteasomal-specific degradation contributes to cardiomyopathic phospholamban null mutation. Mol. Med. 27 (1), 102. 10.1186/s10020-021-00362-8 34496741 PMC8425124

[B93] Sanoudou D. Goehringer J. Morales A. (2025). “Genetic counseling for cardiovascular disease: Part B – post-test approaches and Considerations,” in Genetic counseling for cardiovascular disease: Part B – post-test approaches and considerations. Editor Seifi M. (London, United Kingdom: IntechOpen). 10.5772/intechopen.1007942

[B94] Sanoudou D. Kolokathis F. Arvanitis D. Al-Shafai K. Krishnamoorthy N. Buchan R. J. (2015). Genetic modifiers to the PLN L39X mutation in a patient with DCM and sustained ventricular tachycardia? Glob. Cardiol. Sci. Pract. 2015 (2), 29. 10.5339/gcsp.2015.29 26535225 PMC4614339

[B95] Santos D. G. Medeiros A. Brum P. C. Mill J. G. Mansur A. J. Krieger J. E. (2009). No evidence for an association between the -36A>C phospholamban gene polymorphism and a worse prognosis in heart failure. BMC Cardiovasc Disord. 9, 33. 10.1186/1471-2261-9-33 19638213 PMC2734742

[B96] Schmitt J. P. Ahmad F. Lorenz K. Hein L. Schulz S. Asahi M. (2009). Alterations of phospholamban function can exhibit cardiotoxic effects independent of excessive sarcoplasmic reticulum Ca2+-ATPase inhibition. Circulation 119 (3), 436–444. 10.1161/CIRCULATIONAHA.108.783506 19139388

[B97] Schmitt J. P. Kamisago M. Asahi M. Li G. H. Ahmad F. Mende U. (2003). Dilated cardiomyopathy and heart failure caused by a mutation in phospholamban. Science 299 (5611), 1410–1413. 10.1126/science.1081578 12610310

[B98] Sel K. Osman D. Zare F. Masoumi Shahrbabak S. Brattain L. Hahn J. O. (2024). Building digital twins for cardiovascular health: from principles to clinical impact. J. Am. Heart Assoc. 13 (19), e031981. 10.1161/JAHA.123.031981 39087582 PMC11681439

[B99] Sharma P. Ignatchenko V. Grace K. Ursprung C. Kislinger T. Gramolini A. O. (2010). Endoplasmic reticulum protein targeting of phospholamban: a common role for an N-terminal di-arginine motif in ER retention? PLoS One 5 (7), e11496. 10.1371/journal.pone.0011496 20634894 PMC2901339

[B100] Simmerman H. K. Jones L. R. (1998). Phospholamban: protein structure, mechanism of action, and role in cardiac function. Physiol. Rev. 78 (4), 921–947. 10.1152/physrev.1998.78.4.921 9790566

[B101] Soukarieh O. Meguerditchian C. Proust C. Aissi D. Eyries M. Goyenvalle A. (2022). Common and rare 5'UTR variants altering upstream open reading frames in cardiovascular genomics. Front. Cardiovasc Med. 9, 841032. 10.3389/fcvm.2022.841032 35387445 PMC8977850

[B102] Sousa A. Canedo P. Azevedo O. Lopes L. Pinho T. Baixia M. (2019). Molecular characterization of Portuguese patients with dilated cardiomyopathy. Rev. Port. Cardiol. Engl. Ed. 38 (2), 129–139. 10.1016/j.repc.2018.10.010 30871747

[B103] Stege N. M. de Boer R. A. Makarewich C. A. van der Meer P. Sillje H. H. W. (2024). Reassessing the Mechanisms of PLN-R14del Cardiomyopathy: From Calcium Dysregulation to S/ER Malformation. JACC Basic Transl. Sci. 9 (8), 1041–1052. 10.1016/j.jacbts.2024.02.017 39297138 PMC11405888

[B104] Stege N. M. Eijgenraam T. R. Oliveira Nunes Teixeira V. Feringa A. M. Schouten E. M. Kuster D. W. D. (2023). DWORF Extends Life Span in a PLN-R14del Cardiomyopathy Mouse Model by Reducing Abnormal Sarcoplasmic Reticulum Clusters. Circ. Res. 133 (12), 1006–1021. 10.1161/CIRCRESAHA.123.323304 37955153 PMC10699510

[B105] Tabata T. Kuramoto Y. Ohtani T. Miyawaki H. Miyashita Y. Sera F. (2021). Phospholamban p.Arg14del Cardiomyopathy: A Japanese Case Series. Intern Med. 61 (13), 1987–1993. 10.2169/internalmedicine.8594-21 34924461 PMC9334245

[B106] Tada M. Toyofuku T. (1998). Molecular regulation of phospholamban function and expression. Trends Cardiovasc Med. 8 (8), 330–340. 10.1016/s1050-1738(98)00032-2 14987547

[B107] Tada M. Yabuki M. Toyofuku T. (1998). Molecular regulation of phospholamban function and gene expression. Ann. N. Y. Acad. Sci. 853, 116–129. 10.1111/j.1749-6632.1998.tb08261.x 10603941

[B108] Toyoshima C. Asahi M. Sugita Y. Khanna R. Tsuda T. MacLennan D. H. (2003). Modeling of the inhibitory interaction of phospholamban with the Ca2+ ATPase. Proc. Natl. Acad. Sci. U. S. A. 100 (2), 467–472. 10.1073/pnas.0237326100 12525698 PMC141018

[B109] Truszkowska G. T. Bilinska Z. T. Kosinska J. Sleszycka J. Rydzanicz M. Sobieszczanska-Malek M. (2015). A study in Polish patients with cardiomyopathy emphasizes pathogenicity of phospholamban (PLN) mutations at amino acid position 9 and low penetrance of heterozygous null PLN mutations. BMC Med. Genet. 16, 21. 10.1186/s12881-015-0167-0 25928149 PMC4421997

[B110] Vafiadaki E. Glijnis P. C. Doevendans P. A. Kranias E. G. Sanoudou D. (2023). Phospholamban R14del disease: the past, the present and the future. Front. Cardiovasc Med. 10, 1162205. 10.3389/fcvm.2023.1162205 37144056 PMC10151546

[B111] Vafiadaki E. Haghighi K. Arvanitis D. A. Kranias E. G. Sanoudou D. (2022). Aberrant PLN-R14del Protein Interactions Intensify SERCA2a Inhibition, Driving Impaired Ca(2+) Handling and Arrhythmogenesis. Int. J. Mol. Sci. 23 (13), 6947. 10.3390/ijms23136947 35805951 PMC9266971

[B112] Vafiadaki E. Kranias E. G. Eliopoulos A. G. Sanoudou D. (2024). The phospholamban R14del generates pathogenic aggregates by impairing autophagosome-lysosome fusion. Cell Mol. Life Sci. 81 (1), 450. 10.1007/s00018-024-05471-1 39527246 PMC11554986

[B113] Vafiadaki E. Papalouka V. Arvanitis D. A. Kranias E. G. Sanoudou D. (2009). The role of SERCA2a/PLN complex, Ca(2+) homeostasis, and anti-apoptotic proteins in determining cell fate. Pflugers Arch. 457 (3), 687–700. 10.1007/s00424-008-0506-5 18415121

[B114] van der Zwaag P. A. van Rijsingen I. A. Asimaki A. Jongbloed J. D. van Veldhuisen D. J. Wiesfeld A. C. (2012). Phospholamban R14del mutation in patients diagnosed with dilated cardiomyopathy or arrhythmogenic right ventricular cardiomyopathy: evidence supporting the concept of arrhythmogenic cardiomyopathy. Eur. J. Heart Fail 14 (11), 1199–1207. 10.1093/eurjhf/hfs119 22820313 PMC3475434

[B115] van Drie E. Taal S. E. L. Schmidt A. F. Verstraelen T. E. de Brouwer R. Schoormans D. (2023). Influence of stressful life events and personality traits on PLN cardiomyopathy severity: an exploratory study. Europace 26 (1), euad368. 10.1093/europace/euad368 38206619 PMC10783237

[B116] van Lint F. H. M. Hassanzada F. Verstraelen T. E. Wang W. Bosman L. P. van der Zwaag P. A. (2023). Exercise does not influence development of phenotype in PLN p.(Arg14del) cardiomyopathy. Neth Heart J. 31 (7-8), 291–299. 10.1007/s12471-023-01800-4 37474840 PMC10400740

[B117] van Rijsingen I. A. van der Zwaag P. A. Groeneweg J. A. Nannenberg E. A. Jongbloed J. D. Zwinderman A. H. (2014). Outcome in phospholamban R14del carriers: results of a large multicentre cohort study. Circ. Cardiovasc Genet. 7 (4), 455–465. 10.1161/CIRCGENETICS.113.000374 24909667

[B118] Verstraelen T. E. van Lint F. H. M. Bosman L. P. de Brouwer R. Proost V. M. Abeln B. G. S. (2021). Prediction of ventricular arrhythmia in phospholamban p.Arg14del mutation carriers-reaching the frontiers of individual risk prediction. Eur. Heart J. 42 (29), 2842–2850. 10.1093/eurheartj/ehab294 34113975 PMC8325776

[B119] Verstraelen T. E. van Lint F. H. M. de Brouwer R. Proost V. M. van Drie E. Bosman L. P. (2025). Age-related penetrance of phospholamban p.Arg14del cardiomyopathy. Eur. J. Heart Fail. 10.1002/ejhf.3672 PMC12803612 40264254

[B120] Vicente M. Salgado-Almario J. Valiente-Gabioud A. A. Collins M. M. Vincent P. Domingo B. (2022). Early calcium and cardiac contraction defects in a model of phospholamban R9C mutation in zebrafish. J. Mol. Cell Cardiol. 173, 127–140. 10.1016/j.yjmcc.2022.10.005 36273660

[B121] Vostrikov V. V. Soller K. J. Ha K. N. Gopinath T. Veglia G. (2015). Effects of naturally occurring arginine 14 deletion on phospholamban conformational dynamics and membrane interactions. Biochim. Biophys. Acta 1848 (1 Pt B), 315–322. 10.1016/j.bbamem.2014.09.007 25251363 PMC4258429

[B122] Wagner M. Weber S. El-Armouche A. (2015). Linking superinhibitory PLN mutations to CaMKII activation: a new arrhythmogenic mechanism in genetic DCM? Cardiovasc Res. 107 (1), 5–6. 10.1093/cvr/cvv163 26014576

[B123] Walsh R. Buchan R. Wilk A. John S. Felkin L. E. Thomson K. L. (2017a). Defining the genetic architecture of hypertrophic cardiomyopathy: re-evaluating the role of non-sarcomeric genes. Eur. Heart J. 38 (46), 3461–3468. 10.1093/eurheartj/ehw603 28082330 PMC5837460

[B124] Walsh R. Thomson K. L. Ware J. S. Funke B. H. Woodley J. McGuire K. J. (2017b). Reassessment of Mendelian gene pathogenicity using 7,855 cardiomyopathy cases and 60,706 reference samples. Genet. Med. 19 (2), 192–203. 10.1038/gim.2016.90 27532257 PMC5116235

[B125] Weber D. K. Reddy U. V. Robia S. L. Veglia G. (2024). Pathological mutations in the phospholamban cytoplasmic region affect its topology and dynamics modulating the extent of SERCA inhibition. Biochim. Biophys. Acta Biomembr. 1866 (7), 184370. 10.1016/j.bbamem.2024.184370 38986894 PMC11457527

[B126] Weber D. K. Reddy U. V. Wang S. Larsen E. K. Gopinath T. Gustavsson M. B. (2021). Structural basis for allosteric control of the SERCA-Phospholamban membrane complex by Ca(2+) and phosphorylation. Elife 10, e66226. 10.7554/eLife.66226 33978571 PMC8184213

[B127] Whiffin N. Karczewski K. J. Zhang X. Chothani S. Smith M. J. Evans D. G. (2020). Characterising the loss-of-function impact of 5' untranslated region variants in 15,708 individuals. Nat. Commun. 11 (1), 2523. 10.1038/s41467-019-10717-9 32461616 PMC7253449

[B128] Wilde A. A. M. Semsarian C. Marquez M. F. Shamloo A. S. Ackerman M. J. Ashley E. A. (2022). European heart Rhythm association (EHRA)/Heart Rhythm society (HRS)/Asia pacific heart Rhythm society (APHRS)/Latin American heart Rhythm society (LAHRS) expert consensus statement on the state of genetic testing for cardiac diseases. Europace 24 (8), 1307–1367. 10.1093/europace/euac030 35373836 PMC9435643

[B129] Xu J. Li Z. Ren X. Dong M. Li J. Shi X. (2015). Investigation of pathogenic genes in Chinese sporadic hypertrophic cardiomyopathy patients by whole exome sequencing. Sci. Rep. 5, 16609. 10.1038/srep16609 26573135 PMC4647833

[B130] Yabuki M. Toyofuku T. Otsu K. Nishida M. Kuzuya T. Hori M. (1998). Involvement of NF-Y in transcriptional regulation of the phospholamban gene. Eur. J. Biochem. 258 (2), 744–751. 10.1046/j.1432-1327.1998.2580744.x 9874243

[B131] Yu Q. Barndt R. J. Shen Y. Sallam K. Tang Y. Chan S. Y. (2024). Mitigation of stress-induced structural remodeling and functional deficiency in iPSC-CMs with PLN R9C mutation by promoting autophagy. bioRxiv. 10.1101/2024.04.17.589921

[B132] Zhou T. Li J. Zhao P. Liu H. Jia D. Jia H. (2015). Palmitoyl acyltransferase Aph2 in cardiac function and the development of cardiomyopathy. Proc. Natl. Acad. Sci. U. S. A. 112 (51), 15666–15671. 10.1073/pnas.1518368112 26644582 PMC4697436

